# Natural history, prognostic factors and patient perceived response to treatment in chronic spontaneous urticaria

**DOI:** 10.1186/s13223-020-00459-5

**Published:** 2020-07-15

**Authors:** Peter Stepaniuk, Manstein Kan, Amin Kanani

**Affiliations:** Division of Allergy and Immunology, Department of Medicine, University of British Columbia, Vancouver, BC Canada

**Keywords:** Chronic spontaneous urticaria, Natural history, Prognostic factors, Humanistic burden, Alternative therapy, Autoimmune, Thyroid, Therapeutic response

## Abstract

**Background:**

Although the diagnosis and management of chronic spontaneous urticaria (CSU) is well documented in the literature, some aspects of the disease remain unclear. We aimed to further describe the natural history, prognostic factors, humanistic burden and uptake of traditional and alternative therapies in patients with CSU.

**Methods:**

This was a prospective, cross-sectional analysis at a single centre. We reviewed patient medical records and conducted a survey in patients with CSU.

**Results:**

72 patients participated in the study with a median duration of CSU of 48 months. 30% of patients had symptoms that resolved in under 2 years with these patients trending towards an older age of onset of CSU (48 ± 17 years). 16% of patients had symptoms lasting 10 years or longer with these patients trending towards a younger age of onset (22 ± 16 years). Patients with a relapsing/remitting disease course (31%) and those with co-existing angioedema (57%) trended towards a longer median duration of CSU (96 and 50 months respectively) and were observed to have a higher proportion of patients reporting CSU duration of 10 years or longer (33% and 25%, p = 0.033 and p = 0.036 respectively). Patients with co-existing autoimmune/thyroid disease (19%) trended towards a shorter median duration of CSU (37 months). 54 patients (75%) reported sleep disturbance and 29 patients (43%) required emergency room visit(s) for symptomatic control. 84% of patients who trialed second generation antihistamines reported a response to treatment, while 73% of patients who trialed omalizumab reported a response to treatment. Patients using alternative medicine such as acupuncture, traditional Chinese medicine and naturopathic medicine had lower reported response rates (20–29%) to treatment.

**Conclusions:**

The natural history of CSU may be longer than previously reported with our study finding a median duration of symptoms of nearly 4 years with one-third of patients reporting a relapsing/remitting disease course. Younger age of onset, a relapsing/remitting disease course and angioedema may predict a longer duration of CSU, whereas older age of onset and co-existing autoimmune/thyroid disease may predict a shorter duration of CSU. Reported symptomatic benefit was higher from guidelines based pharmacologic therapy versus various alternative medicines.

## Background

Chronic spontaneous urticaria (CSU) is defined as the presence of urticaria with or without angioedema that has been continuous or intermittent for at least 6 weeks [1]. It can often cause symptoms that significantly impact quality of life for those affected. While the diagnosis and treatment of CSU has been well documented in the literature, the natural history, long term outcomes and prognostic factors are poorly understood for this condition. There is also limited information on the use of complementary and alternative medicine in patients with CSU as well as the perceived benefit when compared with standard pharmacologic therapy. The goal of this study was to answer these questions and further report on the burden of disease for both the individual and the health care system, particularly in Canada.

A recent cross sectional analysis has estimated that CSU affects about 0.23% of the population in the United States, with women more than twice as likely to have the condition relative to men [2]. CSU affects individuals across their entire adult lifespan, with a slightly higher prevalence in patients in their fifth and sixth decades of life [2]. The prevalence of disease among various ethnicities is also felt to be fairly comparable [2]. Few studies have evaluated the natural history of CSU with conflicting results about long term remission rates [3–5] Most of these studies that have analyzed the natural history have been designed as single centered studies with limited long term follow up of patients. The general consensus is that about one-third to one-half of patients with CSU will have remission of their disease within 1 year [3, 6, 7]. However, longer term remission rates are less consistent with estimations of ongoing disease at 5 years ranging from 15 to 70% [3, 8]. More recent studies that have looked at remission rates in children, estimate that remission rates are low and are only about 10.3% per year [9]. The presence of angioedema is believed to predict CSU disease duration and severity [3]. There has also been some evidence that patients with autoimmune urticaria (based on positive autologous skin serum testing), have a shorter duration of disease and a higher rate of remission [7, 10]. However, other studies have disagreed with this finding, showing no relationship to positive autologous skin serum testing and disease duration/severity [3]. More information is required to better define the long-term prognosis and predictors of severity and duration for patients with CSU.

Several studies have shown that the symptoms of CSU can often have a significant effect on patient well-being, utilization of health care resources and quality of life, particularly sleep and work impairment [11–13]. There are various scoring systems available to assess the burden of disease for patients with CSU. One of the most commonly used is the Urticaria Activity Score (UAS7) which assigns points based on the number of wheals present as well as severity of pruritus, including impact on daily activities and sleep, for seven consecutive days [14]. A recent study that collected patient reported data in the Adelphi Real World 2015 Urticaria Disease Specific Programme reported that the most common symptoms reported by patients with CSU included itch, sleep problems and anxiety/distress which affected 75%, 23% and 18% of patients respectively [15]. Overall however, few studies have evaluated the burden on health care resources. One study from Brazil estimates that patients with CSU have twice the number of physician visits and twice the number of emergency room visits when compared to controls [12]. Similarly, an Argentinian study showed that 7.8% of patients with CSU required emergency room visits to control their symptoms with an average yearly direct cost per patient estimated to be USD $1015 ± $752 [16].

Management guidelines for CSU have recently been developed and accepted by most jurisdictions. These guidelines recommend initial treatment with second generation antihistamines, which can be used up to four times the recommended dose if there is inadequate response to standard dosing [17]. Omalizumab and cyclosporine, added on to treatment with antihistamines, are recommended as second- and third-line treatments respectively. Short courses of steroids may be required for acute exacerbations [1]. First generation antihistamines, H2-receptor antagonists, leukotriene receptor antagonists, and other immunosuppressive drugs including sulfasalazine, dapsone, and intravenous immunoglobulin (IVIG) were previously recommended as add on therapy to those not responding to antihistamines [18]. However, the use of these drugs have recently fallen out of favour due to the more effective and better tolerated medications that are currently available.

Although there are many different forms of complementary and alternative medicine available, their effectiveness in treating CSU is questionable. There has been some studies that looked at acupuncture for global symptom improvement when used in conjunction with pharmacologic therapy however a recently published meta-analysis showed there is overall a low level of evidence for its use [19]. There has also been a few randomized trials that have looked at the use of traditional Chinese medicine as an adjunct to traditional pharmacologic therapy in CSU with varying results [20–23]. However, to our knowledge, there has not been any studies that have evaluated patient uptake of these alternative medicine practices and their perceived benefit.

## Methods

### Study design

This was a prospective cross-sectional, single centre study that took place at a private allergy/immunology clinic in Vancouver, British Columbia, Canada. Potential participants were identified by diagnostic code (CSU) in the clinic’s electronic medical record from 2013 to 2015. Each file was manually reviewed by the investigators for inclusion in the study (see criteria below). Relevant medical data obtained from the medical chart included patient age, gender, baseline medical history including medical comorbidities, duration and severity of CSU, prior treatments, relevant co-morbid inducible urticarias, prescribed treatments, and laboratory results (if applicable). Data was collated in an electronic spreadsheet with patients identified by a unique participant identification number.

Eligible participants who met the inclusion criteria for the study (see below) were sent a package in the mail which included a letter of initial contact outlining the purpose of the study and an informed consent document. The letter of initial contact stated that eligible participants would be contacted in 4–6 weeks for a follow-up telephone survey if they had signed and returned the consent form to the investigator’s clinic and agreed participate in the study. If patients declined participation, no further contact was made. If patients did not respond to the initial package, a follow-up phone call was made to further describe the study and answer any questions. Some eligible participants were also approached at follow-up visits, as opposed to communication through letter mail, if they had a follow-up visit in the clinic within 4–6 weeks. A telephone survey with a prepared oral script took place 4–6 weeks after initial contact to gather participant-reported data (Additional file 1: Figure S1). Patients that had a follow-up clinic visit scheduled within 4–6 weeks from initial contact had the option to complete the survey in clinic as opposed to over the telephone if they preferred. The survey asked questions regarding ethnicity, onset of symptoms, number of days per month with urticarial lesions present (either continuous or intermittent), length of total disease course (including remission if applicable), relapse rates and duration, associated angioedema, identification of specific triggers (not confirmed on provocation testing), self-reported severity of disease (including sleep disturbance), treatment(s) received with response rates and alternative treatments sought with their perceived response. Although not standardized, we did record relevant laboratory findings in our patients with CSU. The ordering of these blood tests was at the discretion of the ordering attending physician and based on the patient’s clinical history and physical exam findings. Not all patients had laboratory results to review and additionally, not all patients with laboratory results had the same tests ordered.

### Study population and data analysis

To be included in the study, patients must have met the clinical diagnostic criteria for CSU, defined as recurrent pruritic urticarial erythematous wheals, with each lesion lasting less than 24 h, with continuous or intermittent symptoms persisting for 6 weeks or longer with no external trigger identified. This included patients with co-existing inducible urticarias. Patients with CSU presenting solely with angioedema in the absence of urticaria were excluded from the study. Patients with both active and resolved disease were included in the analysis, and only patients with resolved disease were used when analyzing the duration of disease. Patients also must have been aged 19 or older, able to provide consent to participate in the study and have been willing to participate and complete the patient surveys. Patients were excluded from the study if they were unable to provide informed consent or if they were unwilling to complete the patient questionnaire. Data obtained from the medical chart and patient survey was entered, stored and analyzed in an electronic spreadsheet by unique patient identifier. Analysis of data was performed by the authors using the electronic spreadsheet functions. Two-tailed statistical analysis were performed to evaluate for differences of study subgroups to determine significance using electronic software.

## Results

### Patient demographics and duration of disease

**Fig. 1:**
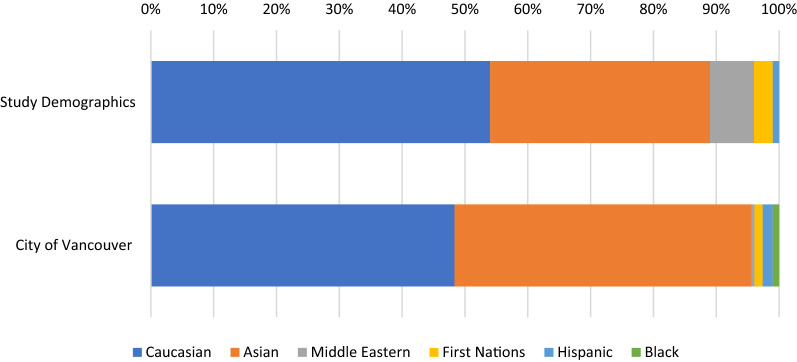
Comparison of ethnic background of study population verses city of Vancouver (%). Proportion of patients in study self-identifying as particular ethnic background compared to ethnic background of city of study (Vancouver, BC) based on recent Canadian census [24]

**Fig. 2:**
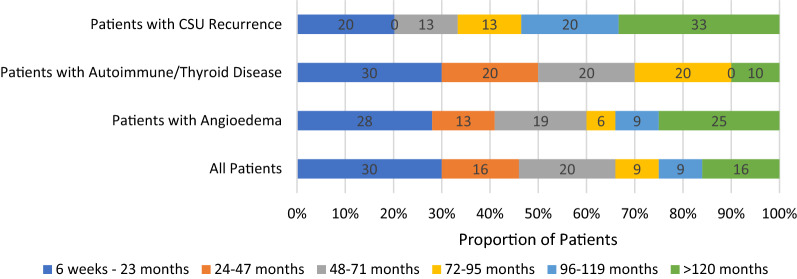
Proportional duration of symptoms in patients with resolved CSU (%). Self-reported duration of symptoms of all patients in study with resolved disease (*n *= 56) compared to patients with CSU recurrence (*n *= 15), those with co-existing angioedema (*n *= 32) and to patients with co-existing thyroid and/or autoimmune disease (*n *= 10)

A total of 72 patients who were eligible for the study agreed to participate and completed the patient survey. 59 (82%) of the patients were female (Table 1). Slightly more than half of the patients were Caucasian (54%), with the reminder identifying as Asian (35%), Middle Eastern (7%) First Nations (3%) and Hispanic (1%). (Figure 1). For comparison, the demographics of the city of Vancouver, British Columbia, Canada (location of study) is also shown [24]. The median age of onset was 43 ± 17 years old. 16 patients (22%) had active disease at the time of data collection and 56 patients (78%) indicated that their CSU had resolved. The median duration of urticaria in patients with resolved CSU was 48 months with a range of 2 to 204 months. Of those with resolved disease, 17 patients (30%) had urticaria that resolved within 2 years, while 9 patients (16%) had CSU that lasted 10 years or longer (Table 2 and Fig. 2). Males with resolved CSU reported a median duration of disease of 33 months whereas females reported a median duration of 48 months. 5 males (45%) and 12 females (27%) had resolution of their CSU within 2 years (p = 0.226). The duration of disease was otherwise similar for males and females (Table 2). Patients with resolved CSU and disease resolution within 2 years had a median age of CSU onset of 48 ± 17 years, and those with disease that lasted 10 years or longer had a median age of CSU onset of 22 ± 16 years. Patients also reported urticarial lesions present for a mean of 21 ± 9 days per month. One patient could not specify on their duration of symptoms, and were excluded from this value.

**Table 1:** Demographics, triggers and associated conditions of patients with CSU

	Number of patients (%) *n* = 72
Sex	
Male	13 (18)
Female	59 (82)
Ethnicity	
Caucasian	39 (54)
Asian	25 (35)
Middle Eastern	5 (7)
First Nations	2 (3)
Hispanic	1 (1)
Recurrence of symptoms after initial remission	22 (31)
Angioedema	41 (57)
Patient reported provoking factors	
Dermatographia	34 (47)
Stress	27 (38)
Heat	25 (35)
Cold	10 (14)
Foods	8 (11)
NSAIDs	8 (11)
Exercise	7 (10)
Alcohol	5 (7)
Sunlight	2 (3)
Sleep disturbance	54 (75)
Emergency room visits	29 (43)
Autoimmune disease	14 (19)
Hypothyroidism	6 (8)
Rheumatoid arthritis	5 (7)
Crohn’s disease	1 (1)
Vitiligo	1 (1)
Celiac disease	1 (1)
HLA-B27 Spondyloarthopathy	1 (1)
Drug allergy	22 (31)
Family history	10 (14)

**Table 2:** Duration of CSU in patients with resolved disease and subgroups

Duration of CSU	Median Duration of CSU (months)	Mean Duration of CSU (months)	6 weeks–23 months	24–47 months	48–71 months	72–95 months	96–119 months	120 months and greater
All Patients with Resolved CSU *n *= 56 (%)	48	61 ± 56	17 (30)	9 (16)	11 (20)	5 (9)	5 (9)	9 (16)
Males *n *= 11 (%)	33	51 ± 61	5 (45)	1 (9)	2 (18)	1 (9)	0 (0)	2 (18)
Females *n *= 45 (%)	48	63 ± 55	12 (27)	8 (18)	9 (20)	4 (9)	5 (11)	7 (16)
Patients with CSU recurrence *n *= 15 (%)	96	101 ± 68	3 (20)	0 (0)	2 (13)	2 (13)	3 (20)	5 (33)
Patients without CSU recurrence *n *= 41 (%)	36	46 ± 43	14 (34.1)	9 (22)	9 (22)	3 (7)	2 (5)	4 (10)
Patients with angioedema *n *= 32 (%)	50	51 ± 13	9 (28)	4 (13)	6 (19)	2 (6)	3 (9)	8 (25)
Patients without angioedema *n *= 24 (%)	35	46 ± 44	8 (33)	5 (21)	5 (21)	3 (13)	2 (8)	1 (4)
Patients with thyroid and/or autoimmune disease *n *= 10 (%)	37	51 ± 52	3 (30)	2 (20)	2 (20)	2 (20)	0 (0)	1 (10)
Patients without thyroid or autoimmune disease *n *= 46 (%)	48	61 ± 57	14 (30)	7 (15)	9 (20)	3 (7)	5 (11)	8 (17)

### Patients with relapsing/remitting course

22 patients (31%) reported a recurrence of their disease after initial remission with a mean length of remission of 21 ± 10 months. Those with disease recurrence had a similar median age of onset of CSU of 42.5 years compared to 44.5 years in those patients denying CSU recurrence. 3 males (23%) and 19 females (32%) reported CSU recurrence, with no statistical difference (p = 0.516). Of those with resolved CSU, 15 patients (27%) reported a disease recurrence, with a total median duration of disease of 96 months (including disease remission time). 5 of these patients (33%) reported a disease duration of 10 years or longer, compared to 4 patients (10%) who had resolution of their CSU and denied a recurrence of disease (p = 0.033) (Table 2 and Fig. 2).

### Angioedema

41 patients (57%) had associated angioedema and of those patients with resolved CSU, 32 patients (57%) had associated angioedema with their median duration of CSU lasting 50 months. 8 patients (25%) had symptoms lasting 10 years or longer compared to only 1 patient (4%) who did not have angioedema (p = 0.036) (Table 2 and Fig. 2). 36 of the 41 patients who experienced angioedema reported that they had an average of 5 ± 7 episodes per month. Five patients who experienced angioedema, were unable to clearly quantify the frequency of their episodes. Of the 22 patients that reported a recurrence of their CSU, 14 patients had associated angioedema (64%), whereas of the 50 patients that did not report disease recurrence, 27 patients (54%) had angioedema (p = 0.447).

### Provoking factors

**Fig. 3:**
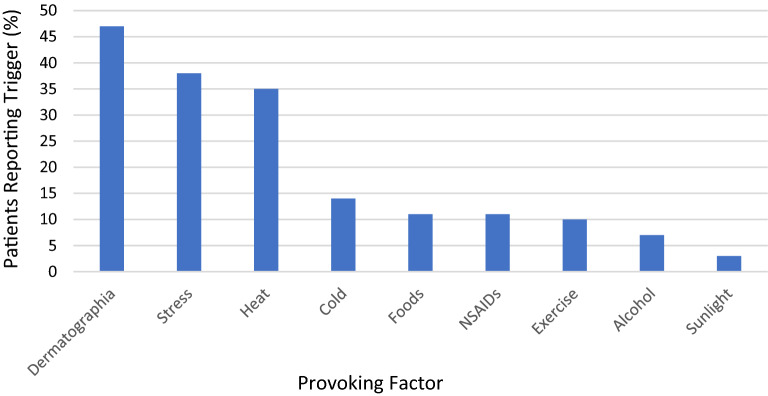
Patient reported provoking factors for CSU. Proportion of patients reporting a trigger for their urticaria. Some patients reported multiple triggers. Triggers were not verified on provocation testing

A significant number of patients reported provoking factors for their urticaria, including dermatographia (47%), stress (38%), heat (35%), and cold (14%). Other triggers reported by patients included foods (11%), nonsteroidal anti-inflammatory drugs (NSAIDs) (11%), exercise (10%), alcohol (7%) and sunlight (3%) (Fig. 3). Physical triggers were considered to be present based on patient perception as indicated in their survey and were not verified on provocation testing.

### Co-morbid autoimmune and allergic diseases

A total of 14 patients (19%) reported co-existing thyroid and/or autoimmune disease, with hypothyroidism (8%) being most common. Other reported autoimmune diseases included rheumatoid arthritis (7%), Crohn’s disease (1%), vitiligo (1%), celiac disease (1%) and HLA-B27 spondyloarthopathy (1%) (Table 1). A few patients had multiple co-existing autoimmune diseases. Of those with resolved CSU, 10 patients (18%) reported a history of autoimmune disease and had a median duration of symptoms lasting 37 months. Three patients (30%) had CSU that resolved within 2 years and one patient (10%) had CSU that lasted 10 years or longer. These findings were not statistically significant (Table 2 and Fig. 2). Of the 22 patients that reported a recurrence of their CSU, 3 patients (14%) had associated autoimmune and/or thyroid disease, whereas 11 patients (22%) without CSU recurrence had autoimmune and/or thyroid disease (p = 0.407). 22 patients (31%) reported co-existing drug allergies and 10 patients (14%) reported a family history of CSU. (Table 1).

### Quality of life indicators

54 patients (75%) reported that they had disrupted sleep due to their urticaria and 29 patients (43%) presented to the emergency room for uncontrollable symptoms of their urticaria. Of note, five patients did not identify if they visited an emergency room for their urticaria (Table 1). 47 females (80%) reported sleep disturbance from their CSU, comparted to only 7 males (54%) (p = 0.512). Duration of disease in those with resolved CSU did not appear to influence either sleep disturbance or emergency room visits. 20 patients (91%) with CSU recurrence reported sleep disturbance, compared to only 34 patients (68%) without CSU recurrence (p = 0.038). There was no statistically significant difference in rates of sleep disturbance or emergency room visits in patients with angioedema or autoimmune/thyroid disease (Table 3).

**Table 3:** Proportion of CSU patients reporting quality of life impairment stratified by disease characteristics

	Patients reporting sleep disturbance	Patients reporting emergency room visits
Males *n *= 13 (%)	7 (54)	5 (38)
Females *n *= 59 (%)	47 (80)	24 (41)
Duration of CSU *n *= 56 (%)		
6 weeks–23 months *n *= 17	13 (76)	4 (24)
24–47 months *n *= 9	7 (78)	5 (56)
48–71 months *n *= 11	6 (55)	3 (27)
72–95 months *n *= 5	5 (100)	3 (60)
96–119 months *n *= 5	4 (80)	3 (60)
120 months and greater *n *= 9	7 (78)	5 (56)
Patients with CSU Recurrence *n *= 22 (%)	20 (91)	9 (41)
Angioedema *n *= 41 (%)	32 (78)	21 (51)
Autoimmune/thyroid disease *n *= 14 (%)	11 (79)	8 (57)

### Pharmacologic treatments, alternative treatments and self-reported response

The most trialed treatment for CSU were second generation antihistamines with 68 patients (94%) reporting their use. Other pharmacologic treatments employed included first generation antihistamines in 51 patients (71%), prednisone in 28 patients (39%), omalizumab in 15 patients (21%), cyclosporine in three patients (4%), montelukast in two patients (3%), intravenous immunoglobulin (IVIG) in one patient (1%) and methotrexate in one patient (1%) (Table 4). Other therapies that were inquired about and were not used included dapsone, hydroxychloroquine and sulfasalazine. Self-reported response to pharmacologic treatment varied, and was 84% for second generation antihistamines, 84% for first generation antihistamines, 75% for prednisone and 73% for omalizumab. Although few patients trialed therapy with cyclosporine and methotrexate, all the patients who used these medications reported a response to therapy. None of the patients that trialed montelukast or IVIG reported response to treatment (Table 4). Some patients reported seeking alternative medicine practitioners to help with management of their CSU. 15 patients sought advice from a naturopath (21%), while five patients visited an acupuncturist (7%) and seven patients sought advice from a traditional Chinese medicine practitioner (10%) (Table 5). Although inquired about, no patients saw a chiropractor, herbalist or massage therapist for their urticaria. Self-reported response to these alternative practices were relatively low with 29% reporting benefit from traditional Chinese medicine, 27% reporting benefit from treatments provided by their naturopath and 20% reporting benefit from acupuncture (Table 5).

**Table 4:** Pharmacologic treatment and patient self-reported response

Medication	Patients reporting having used therapy *n *= 72 (%)	Patients reporting response to treatment (%)
Second generation antihistamines	68 (94)	57 (84)
First generation antihistamines	51 (71)	43 (84)
Prednisone	28 (39)	21 (75)
Omalizumab	15 (21)	11 (73)
Cyclosporine	3 (4)	3 (100)
Monteleukast	2 (3)	0 (0)
IVIG	1 (1)	0 (0)
Methotrexate	1 (1)	1 (100)

**Table 5:** Complimentary/alternative medicine use and patient self-reported response

Complimentary/alternative practitioner	Patients reporting having used therapy *n *= 72 (%)	Patients reporting response to treatment (%)
Acupuncture	5 (7)	1 (20)
Naturopath	15 (21)	4 (27)
Traditional Chinese medicine	7 (10)	2 (29)

We stratified our patient population by response to treatment with second generation antihistamines to see if we could observe any differences in patient characteristics in these two groups (Table 6). Of those that received treatment with second generation antihistamines, 48 females (87%) and 9 males (69%) reported treatment response to antihistamines while 4 males (31%) and 7 females (13%) did not (p = 0.112). Median age of CSU onset was similar between the two groups. There was no association between treatment response with second generation antihistamines and CSU recurrence, angioedema or autoimmune/thyroid disease (p = 0.418, p = 0.834 and p = 0.159 respectively). Patients that did not report response to treatment with second generation antihistamines trended towards reporting higher rates of sleep disturbance (91%) and emergency room visits (64%) than those that did respond to treatment with antihistamines (74% and 37% respectively), but this was not statistically significant (p = 0.219 and p = 0.099 respectively). There was no difference observed in uptake of alternative therapies in the two groups.

**Table 6:** Demographics and response to therapies of CSU patients stratified by recurrence of disease and response to second generation antihistamines

	Patients reporting response to treatment with second generation antihistamines *n* = 57 (%)	Patients reporting no response to treatment with second generation antihistamines *n* = 11 (%)
Sex		
Male	9 (16)	4 (36)
Female	48 (84)	7 (64)
Median age of onset of CSU (years)	44	42
CSU recurrence	14 (25)	4 (36)
Angioedema	33 (58)	6 (55)
Autoimmune/thyroid disease	10 (18)	4 (36)
Sleep disturbance	42 (74)	10 (91)
Emergency room visits	21 (37)	7 (64)
Alternative therapies		
Visited acupuncturist	5 (9)	0 (0)
Visited naturopath	13 (23)	2 (18)
Visited traditional Chinese practitioner	6 (11)	1 (9)

### Laboratory findings

Standardized laboratory investigations were not part of this protocol. However, we did observe that of 42 patients that had blood work including complete blood counts, 2 of them had eosinophilia (> 0.5 × 10^9^ /L). 27 patients had evaluation for C-reactive protein (CRP) and it was elevated in 8 patients (> 4.8 mg/L). 38 patients had testing for thyroid peroxidase antibodies (anti-TPO) and it was positive in 4 patients (> 35 IU/ml). 23 patients had laboratory testing for immunoglobulin E (IgE), and it was elevated in 11 patients (> 430 μg/ml).

## Discussion

Similar to previous studies, we found that CSU is a disease that disproportionately affects women and has a median age of onset in the fifth decade of life. Previous studies have suggested that the prevalence of CSU is similar among different ethnicities [2]. We found approximately half of our study population was Caucasian and about one-third were of Asian descent. This is fairly close to the overall demographics the total population (city of Vancouver) and supports the idea that prevalence rates are similar among different ethnicities [24]. There was a slightly higher proportion of Caucasians and those of Middle Eastern ethnicity noted (relative to our city’s demographics) however, due to the study size, it is difficult to make conclusive statements. In patients with resolved disease, 30% of patients had urticaria that resolved within 2 years and 16% of patients had their urticaria last 10 years or longer with a median duration of 48 months (4 years). It should be noted that our study population is likely biased to patients with a more severe/refractory course due to the referral process, and the fact that we are a specialist clinic. We noticed a large variability in the reported duration of disease, which ranged from 2 to 204 months. A handful of patients with a very long disease course affected our statistical analysis as evidenced by the significant difference between our calculated median and mean duration of disease as well as our large standard deviation (Table 2). We observed that males trended to have more rapid CSU resolution (within 2 years), but this was not statistically significant. The median age of onset of CSU was also older (48 ± 17 years) in patients who had disease resolution within 2 years compared to patients who had CSU lasting 10 years or longer (22 ± 16 years). Due to the large standard deviation, we cannot make definitive statements, however these findings may suggest that younger age of CSU onset is a risk for prolonged disease course.

About one-third of patients had recurrence of their disease after going into remission, with duration of remission lasting nearly 2 years on average and total median duration of disease lasting about 8 years (including remission time). Patients with recurrence of their CSU also had a higher proportion of patients with disease lasting 10 years or longer (33%), compared to those without disease recurrence (10%) (p = 0.033). This is a significant finding as details on long term prognosis of patients with CSU is limited. The natural history of the disease process may be longer than previously reported and include more of a relapsing/remitting course. Multiple previous studies that evaluated the natural history of CSU do not comment on recurrence rates after the initial remission, and have generally only followed patients for a few years after diagnosis [3, 6, 7]. Neither response to second generation antihistamines, co-existing angioedema nor autoimmune/thyroid disease were observed to be predictors of disease recurrence with comparable rates of recurrence in these groups compared to the entire CSU study population. We did observe a higher reported rate of sleep disturbance (91%) in patients with CSU recurrence. There is currently very limited data on predicators of relapse of CSU, and at this time only disease duration and baseline UAS7 are thought to be predictors of relapse, similar to what we observed [25].

Patients with angioedema had a median duration of CSU of 4.2 years and also had a larger proportion of patients with CSU lasting 10 years (25%) compared to those who did not have angioedema (4%) (p = 0.036). Previous studies have also reported that patients with co-existing angioedema are more likely to have a longer disease course [3]. We also found that patients with co-morbid autoimmune or thyroid disease had a median duration of CSU of just over 3 years. However due to the large variability of data and our small sample size, this was not statistically significantly lower than those without co-morbid autoimmune or thyroid disease. Of note, the range of disease length varied widely in all our groups of patients making conclusive statistical analyses difficult in our small study population. Neither angioedema nor co-morbid autoimmune/thyroid disease were associated with differences in quality of life indicators (sleep disturbance or emergency room visits) or with therapeutic response to second generation antihistamines.

The impact on quality of life was observed to be quite high in our patient population, with three-quarters of our patients reporting sleep disturbance due to the symptoms of CSU. This is a higher proportion than what has previously been reported in the literature [15]. This is a significant finding as it emphasises the humanistic burden that CSU can have and the difficulties in controlling symptoms. In addition, 43% of patients in this study reported a need to visit an emergency room for assistance in control of their symptoms. This is also a higher finding than reported in previous studies [16]. We also observed that patients with CSU recurrence reported a higher rate of sleep disturbance (91%) than patients without sleep disturbance (68%) (p = 0.038) which may suggest that patients with recurrent CSU may have more difficult to control symptoms. Again, however our study population is likely to have a more severe disease course than the entire population with CSU as we are a specialist clinic and are more likely to be referred patients with more severe/persistent symptoms. Although CSU can generally be managed in an outpatient setting, it can create a heavy burden on health care resources if inadequately controlled. Overall, analysis of the data from our study emphasized the high direct and indirect costs that CSU can have on both the patient and health care system, and the importance in controlling the disease to mitigate these costs.

There has also been strong links found between CSU and autoimmunity. Another large Israeli population-based study that spanned 17 years and involving thousands of patients diagnosed with CSU found that patients with CSU were observed to have increased odds of co-existing hypothyroidism, hyperthyroidism, and antithyroid antibodies [26]. In addition, female patients with CSU had a significantly higher incidence of rheumatoid arthritis, Sjogren’s syndrome, celiac disease, type 1 diabetes, and systemic lupus erythematous. Most of these autoimmune conditions were diagnosed within the 10 years succeeding the CSU [26]. We found similar findings as 18% of our CSU patients had coexisting autoimmune disease with hypothyroidism and rheumatoid arthritis being most common (8% and 7% respectively). We could not identify any significant differences in our patients with co-morbid autoimmune disease compared to those without co-morbid disease as these two groups had similar rates of recurrence rates, quality of life indicators and response to antihistamine therapy. However, as we only had 14 CSU patients with co-morbid autoimmune thyroid disease, we may have not had a large enough sample size to see statistically significant differences.

The treatment of CSU observed in this study paralleled the recommended published guidelines, with nearly all patients having tried second generation antihistamines followed by first generation antihistamines, prednisone and omalizumab [1]. However, patient reported response rates to these common pharmacologic therapies ranged from 73 to 84%. Omalizumab was approved for treatment of chronic spontaneous urticaria in August 2014 in Canada. As this data was collected shortly after drug approval, the proportion of patients who trailed treatment with omalizumab versus other immunosuppressive medications is likely lower in this study than it would be if the same data was collected today. Although only a few patients had trialed cyclosporine, all of those patients reported relief of their symptoms from its use. There were a smaller number of patients trialed on treatment with methotrexate, IVIG and montelukast and interpretation of response to these medications is limited by the small sample size. These findings suggest that even with treatment regimens that follow consensus guidelines, about one-fifth of patients do not report symptomatic response to treatment. This may explain why some patients seek out complementary and alternative medicine for assistance in relief of symptoms. Unfortunately, for patients that trial alternative medicine, their reported response rates are even lower with only 20–29% of patients reporting benefit in our study. This would be consistent with the inconclusive studies in acupuncture and traditional Chinese medicine [19–23]. Although, 21% of our patients reported visiting a naturopath, we could not find any studies in the literature that have studied the use of and effectiveness of naturopathic therapies in the treatment of CSU. Because of the relatively low response rates to these multiple alternative therapies, their use should be discouraged.

Although this is a relatively small analysis, we feel that this adds to the growing body of evidence about CSU. Limitations of this study include the small sample size and single centre origin which makes generalizability to the general population difficult. In addition, there is likely a component of selection bias as patients were required to volunteer to complete the survey and participate in the study. This may predispose individuals who have had a more difficult time controlling their symptoms agreeing to participate in the study. In addition, our study population is based on referrals to an allergy specialist referral centre and are therefore also more likely to have severe disease.

## Conclusions

CSU has been shown to disproportionately affect females with an average onset of disease in the fifth decade and generally similar rates among various ethnicities. We believe that the natural history of CSU may be longer than previously reported with our study showing a median duration of symptoms of 4 years and one-third of patients having a relapsing/remitting course. Patients with a relapsing/remitting disease course and those with co-existing angioedema trended towards a longer median duration of CSU and were observed to have a higher proportion of patients reporting CSU duration of 10 years or longer. Patients with co-existing autoimmune/thyroid disease trended towards a shorter median duration of CSU. There was also a trend of older age of CSU diagnosis being associated with more rapid CSU resolution (within 2 years) whereas those diagnosed with CSU at a younger age trended towards a longer disease course (10 years or greater). There was a relatively high humanistic burden of disease with 75% of patients reporting inadequate symptom control disrupting their sleep as well as significant utilization of health care resources (i.e. emergency room visits) for patients with uncontrolled symptoms, particularly in those with a relapsing/remitting disease course. Reported symptomatic benefit was higher from guideline based pharmacologic therapy versus various alternative medicines with response rates to antihistamines similar amongst different subgroups of patients. CSU can have a significant impact on quality of life if it is inadequately controlled and even with following guideline-based therapy, symptoms can be difficult to abate in a subset of the population.

## Supplementary information

**Additional file 1:** Figure S1. Study questionnaire provided to patients.

## Data Availability

All data generated or analyzed during this study are included in this published article.

## Abbreviation

IVIG: Intravenous immunoglobulin
