# Supplementary material for: Natural history, prognostic factors and patient perceived response to treatment in chronic spontaneous urticaria
Source: Allergy Asthma Clin Immunol. 2020 Jul 15;16:63. doi: 10.1186/s13223-020-00459-5 (PMC7371813; doi:10.1186/s13223-020-00459-5)
Supplement: Supplementary file 1 — Additional file 1: Figure S1. Study questionnaire provided to patients. [file 13223_2020_459_MOESM1_ESM.docx]

**Figure S1: Study Questionnaire Provided to Patients**

*Natural History, Long Term Outcomes and Prognostic Factors for Patients with Chronic Urticaria (Hives) – Oral Script*

The following questions are to help us better understand the nature of your condition. Please answer the following questions to the best of your ability.

1. When did you first notice (in month and year) your hives? How long did it last (in months)?
2. If not resolved, how long has it been active (in months)?
3. Has your urticaria relapsed? (What year/month and duration of each relapse?)
4. How often do you experience urticaria? (number of days/week)
5. Have you experienced angioedema or swelling of the lip, eyelid or other body parts?
6. Number of angioedema episodes/months?
7. Please indicate whether these trigger/exacerbate your hives: stress, heat, exercise, cold, physical (dermographism), alcohol, sunlight, food, NSAIDs
8. Have you had any treatment for your urticaria? (please circle if you have used any of these medications: Antihistamines (1^st^ generation: hydroxyzine (Atarax®), diphenhydramine (Benadryl®), chlorpheniramine (Chlor-Tripolon®), promethazine (Histantil®), 2^nd^ generation: cetirizine (Reactine®), loratadine (Claritin®), fexofenadine (Allegra®), desloratadine (Aerius®)), dapsone, sulfasalazine, hydroxychloroquine, cyclosporine, tacrolimus, mycophenolate, omalizumab, IV immunoglobulin, prednisone, monteleukast, ranitidine/cimetidine).
9. For each of the medication(s) used in question 8, please indicate whether you responded to the treatment? (Yes, No, Partially)
10. Are you currently taking any of these medications for your urticaria? Please circle from the list of medications below if applicable. If the medication is no shown, please write the name and dose of medication used. (antihistamine, dapsone, sulfasalazine, hydroxychloroquine, cyclosporine, tacrolimus, mycophenolate, omalizumab, IV immunoglobulin, prednisone, monteleukast, ranitidine/cimetidine).
11. Does urticaria bother your sleep?
12. Do you have any (hypo/hyper) thyroid problems, autoimmune disease (i.e. lupus, rheumatoid arthritis) or white patches of skin (vitiligo)?
13. Do you have any drug allergies?
14. Any family history of chronic urticaria?
15. Have you seen any of the following alternative health care providers for your hives (i.e. naturopathy, herbalist, chiropractor, Chinese traditional medicine, massage therapist) and did you respond to treatment?
16. We are investigating the relationship between different factors in chronic urticaria. One factor which we are trying to determine is association with duration of urticaria is ethnicity. What is your ethnic background?
